# Protocol for a national audit on self-reported confidence levels, training requirements and current practice among trainee doctors in the UK: The Trainees Own Perception of Delivery of Care in Diabetes (TOPDOC) Study

**DOI:** 10.1186/1472-6920-10-54

**Published:** 2010-07-27

**Authors:** Jyothis T George, David J McGrane, David Warriner, Kavitha S Rozario, Hermione C Price, Emma G Wilmot, Partha Kar, Edward B Jude, Gerard A McKay

**Affiliations:** 1 The Queen's Medical Research Institute, 47 Little France Crescent, Edinburgh, UK; 2 Inverclyde Royal Hospital, Greenock, UK; 3 Barnsley District General Hospital, Barnsley, UK; 4 Royal Hospital for Sick Children, Edinburgh, UK; 5 Horton Hospital, Banbury, UK; 6 University Hospitals of Leicester, Leicester, UK; 7 Queen Alexandra Hospital, Portsmouth, UK; 8 Tameside General hospital, Ashton-under-Lyne, UK; 9 University Medical Unit, Glasgow Royal Infirmary, Glasgow, UK

## Abstract

**Background:**

As the incidence and prevalence of diabetes increases across the world, resource pressures require doctors without specialist training to provide care for people with diabetes. In the UK, national standards have been set to ensure quality diabetes care from diagnosis to the management of complications. In a multi-centre pilot study, we have demonstrated a lack of confidence among UK trainee doctors in managing diabetes. Suboptimal confidence was identified in a number of areas, including the management of diabetes emergencies. A national survey would clarify whether the results of our pilot study are representative and reproducible.

**Methods/Design:**

**Target cohort**: All postgraduate trainee doctors in the UK.

Domains Studied: The self reported online survey questionnaire has 5 domains: (1) confidence levels in the diagnosis and management of diabetes, (2) working with diabetes specialists, (3) perceived adequacy of training in diabetes (4) current practice in optimising glycaemic control and (5) perceived barriers to seeking euglycaemia.

**Assessment tools**: Self-reported confidence is assessed using the 'Confidence Rating' (CR) scale for trainee doctors developed by the Royal College of Physicians. This scale has four points - ('not confident' (CR1), 'satisfactory but lacking confidence' (CR2), 'confident in some cases (CR3) and 'fully confident in most cases' (CR4).

Frequency of aspects of day-to-day practice is assessed using a six-point scale. Respondents have a choice of 'always' (100%), 'almost always' (80-99%), 'often' (50-79%), 'not very often' (20-49%) and 'rarely' (5-19%) or never (less than 5%).

**Discussion:**

It is anticipated that the results of this national study will clarify confidence levels and current practice among trainee doctors in the provision of care for people with diabetes. The responses will inform efforts to enhance postgraduate training in diabetes, potentially improving the quality of care for people with diabetes.

## Background

Diabetes is one of the greatest challenges facing healthcare systems across the world. As the prevalence of diabetes continues to increase [1] across age groups, doctors without a specialist interest in diabetes are increasingly involved in healthcare provision for individuals with diabetes. Moreover, it is estimated that around a fifth of all hospitalised patients have a diagnosis of diabetes [2], making it likely that doctors specialising in almost all branches of medicine will have patients with diabetes under their care.

In the UK, the National Service Framework (England and Wales) and the Scottish Diabetes Framework lay down standards for diabetes [3]. In the tax-funded National Health Service (NHS) setting in the UK, doctors in postgraduate training provide care for individuals with diabetes in a hospital setting, often supervised and supported by non-specialist doctors or a specialist nurse-led inpatient diabetes service [4]. As providers of current inpatient care and as future general practitioners and specialists, postgraduate trainee doctors have a central role in delivery of diabetes care to national standards.

In a multi-centre pilot cohort of UK trainee doctors, we have demonstrated a lack of confidence in managing diabetes, including the management of diabetes related emergencies [5]. If the findings of our pilot are corroborated at a national level, focused efforts will be needed to address the specific shortfalls in postgraduate training in diabetes. Additionally, a successful survey might act as a template for assessing the confidence and perceived training needs for management of other common medical conditions.

Our aim was to undertake a national audit on self-reported confidence levels, training requirements and current practice among trainee doctors in the UK, auditing adherence to National Service Framework in Diabetes and the Scottish Diabetes Framework. Table 1 summarizes the relevant care standards laid out in these policy documents and the audit questions that arise from these.

**Table 1:** NHS National Service Framework for Diabetes standards relevant to doctors in training and relevant audit questions.

Standard	Audit Questions
**Standard 2: Identification of People with diabetes:** "The NHS will develop, implement and monitor strategies to identify people who do not know they have diabetes."	How confident are doctors in identifying people with diabetes?
**Standard 3: Empowering people with diabetes:** "This will be reflected in an agreed and shared care plan in an appropriate format and language."	How confident are doctors in modifying diabetes pharmacotherapy? How often do they initiate change?
**Standard 4: Clinical care of adults with diabetes:** "All adults with diabetes will receive high-quality care throughout their lifetime, including support to optimise the control of their blood glucose, blood pressure and other risk factors for developing the complications of diabetes."	How often do doctors look for diabetes complications? How confident are doctors in modifying diabetes pharmacotherapy?
**Standard 7: Management of diabetic emergencies:** "The NHS will develop, implement and monitor agreed protocols for rapid and effective treatment of diabetic emergencies by appropriately trained health care professionals".	How confident are doctors in managing diabetic emergencies? What are the training needs of doctors in this area?
**Standard 8: Care of people with diabetes during admission to hospital:** "All children, young people and adults with diabetes admitted to hospital, for whatever reason, will receive effective care of their diabetes."	How confident and trained are doctors in dealing with diabetes in in-patients?
**Standard 10: Detection and management of complications: **"All young people and adults with diabetes will receive regular surveillance for the long-term complications of diabetes."	How confident and trained are doctors in dealing with diabetic complications?

## Methods/design

### Study population

Our target population was all postgraduate trainee doctors (foundation and specialist trainees) practicing in the UK at the time of the survey. There are up to 50,145 training posts in the UK [6], of which some are unfilled [7], and the exact number remains subject to debate. Formal power calculations suggested a minimum target of 248 responses to give 90% power at 0.05% significance between consecutive answers in the six-point rating scale used in the study. Our target was to get responses from 1000 UK trainee doctors across all specialties other than diabetes and endocrinology in this survey, representing around 2% of the total potential study population. Responses from specialist trainees in diabetes and endocrinology will be analyzed separately from the rest of the cohort to avoid bias from the additional diabetes training that these doctors have received. Whilst in the traditional postgraduate training system with parallel recruitment and qualification streams for medical, surgical, psychiatric and other branches of medicine, the current post-graduate training system tries to provide all postgraduate trainees with experience and exposure to as many of these branches as possible. Moreover, trainees from all specialties are pooled together for out-of-hours inpatient care.

### Domains Studied

The following five domains were assessed in our study questionnaire: (1) self reported confidence levels in the diagnosis and management of diabetes, (2) working with diabetes specialists, (3) the adequacy of training in diabetes during and undergraduate and postgraduate training, (4) current practice in seeking glycaemic control and (5) the perceived barriers that prevent trainees from seeking optimum glycaemic control. The survey asked trainees to report their confidence levels in the management of other common medical conditions like chest pain and exacerbation of asthma to clarify whether any lack of confidence and perceived training needs were specific to managing diabetes.

### Assessment tools

To assess self-reported confidence, we chose to use the 'Confidence Rating' (CR) scale used by the Royal College of Physicians in the self-assessment of trainee doctors [8]. The scale has four points - ('not confident' (CR1), 'satisfactory but lacking confidence' (CR2), 'confident in some cases (CR3) and 'fully confident in most cases' (CR4).

To assess the frequency of aspects of day-to-day practice, we chose to use a six-point scale with narrative description in combination with numeric values. Respondents have a choice of 'always' (100%), 'almost always' (80-99%), 'often' (50-79%), 'not very often' (20-49%) and 'rarely' (5-19%) or never (less than 5%).

Spaces were provided for respondents to give 'free-text' comments, which will be subjected to qualitative analysis.

### Validation

The questionnaire has been validated in a four-stage process: Initial review by external experts in the field of diabetes, administration of initial draft on a sample cohort, revision of questionnaire based on feedback and final external review.

### Personal Information

To enable characterization of the respondent population we asked them to provide the following information: (1) year of primary medical qualification, (2) current designation, (3) deanery/region, (4) current and destination specialties and (5) the number of years of full-time postgraduate training respondents have had. Respondents were asked to volunteer their email address to be forwarded the results of the study when the analysis has been completed. They were also asked to provide their General Medical Council registration number, which would be used to validate that a doctor completed the questionnaire and that there was no duplicate submissions.

### Technical design

The study was hosted on a dedicated web portal, http://www.topdocdiabetes.org. Open source software was used with necessary customization to minimize resource utilization. The online version of the questionnaire was compliant with industry standard web programming technologies and subject to best practices in data protection and management. The system logs the location of the respondent computer (IP address) as well as the time and date of submission.

Questions that are 'mandatory' were highlighted to the users and online prompts were provided if any of the mandatory questions were left unanswered.

The survey software provides the principal investigators with national as well as regional breakdown of responses, thus giving the potential to feed back to individuals responsible for diabetes education within each region.

### Recruitment

Recruitment of respondents was done through a multipronged promotional campaign. Press releases were sent to medical news sources (e.g BMA News) and various online doctors fora. Respondents were offered anonymity to encourage participation.

We established a network of regional facilitators to drive recruitment through their local hospital or regional training networks. We were able to offer local facilitators access to data pertinent to their region once the analysis of results is complete. Invitations and promotional materials were sent to all post-graduate training centers in the UK. Incentives were offered in the form of online vouchers on a 'lottery' basis.

With support from Diabetes UK, Association of British Clinical Diabetologists (ABCD), Society for Endocrinology, NHS Diabetes and the Young Diabetologists Forum, we informed diabetes specialists about the study. Through these routes we asked for the assistance of specialists to support local campaigns to recruit participants.

As recruitment is multimodal and with various recruitment strategies likely to overlap, it would be unfeasible to calculate response rate for each method adopted. However, on a national level, a response rate of at least 1 in 50 will achieve our recruitment target.

### Governance

We sought the advice of the Glasgow Royal Infirmary Local Research Ethics Committee as to whether we needed formal ethical approval for this online survey and were advised that it was not required under NHS research governance arrangements. The study is being co-coordinated from a central office with regular updates provided to regional facilitators and study investigators. All data is being held in a secure form and identifiable information will be removed from responses before datasets are shared with statisticians or other research support staff.

### Funding

Funding was secured by a successful competitive bid for the 2008 ABCD audit award. Initial pump-priming funding was provided by Sanofi-Aventis as an educational grant.

### Reporting of results and statistical analysis

We will submit the results of the survey for publication in general medical journals. Self-reported answers will be tabulated and expressed as number (n) and in percentages rounded off to the next percentage point. A p value less than 0.05 will be considered statistically significant.

In addition to overall results, we will stratify responses in each of the domains by year of primary medical qualification, and years of post-graduate medical training to identify trends. Regional facilitators will be provided with summary of results from their region to enable comparison with national average. Where sub-group analyses are under-powered, this will be made clear in relevant manuscripts.

Qualitative analysis of free text from our pilot data identified three broad themes as barriers to optimization of glycaemic control by post-graduate trainees [9]. Firstly, many trainee doctors perceive diabetes as a clinical area that should be managed by specialists. Secondly, routine involvement by specialist teams is felt to hamper training and management opportunities. Thirdly, the organisation of inpatient medical care where a series of junior (and senior) doctors are involved in the management is felt to present many challenges. Similar thematic assessments will be carried out with free-text comments from this study.

### Implementation and Dissemination

We aim to disseminate finding of this audit as widely as possible. Our regional facilitators will present the findings at their regional and local meetings while the steering committee will ensure visibility at a national level. We aim to publish our findings in general and specialist medical journals. We will also forward the results to key stakeholders including ABCD, Diabetes UK, Conference of Postgraduate Deans, deaneries, foundation schools and the national clinical director for diabetes.

## Discussion

The prevalence of diabetes has increased and therapeutic options have become complex. This study assesses the readiness of junior doctors to cope with this. In our multi-centre pilot cohort of UK trainee doctors, we demonstrated a lack of confidence in managing aspects of diabetes care, including the management of diabetes emergencies. This national survey draws on a much larger and more representative sample, enabling a better understanding of confidence levels and current practice among trainee doctors in the provision of care for people with diabetes. If the findings of our pilot are corroborated at a national level, the responses might allow for focused efforts to be made to address specific needs for postgraduate training in diabetes. We anticipate that this could be done at a local and national level. This will ensure patients with diabetes in the UK are managed to the highest standards. In addition to disseminating the results widely our study methods might act as a template for other specialists who may want to establish trainees confidence and perceived training needs in managing diseases within their specialty remit.

## Abbreviations

ABCD: Association of British Clinical Diabetologists; NHS: National Health Service; TOPDOC Diabetes: Trainees Own Perception of Delivery of Care in Diabetes.

## Competing interests

This study received an educational grant from Sanofi-Aventis to prime the study and prepare the successful bid for the 2008 ABCD audit award. This award is sponsored by Eli Lilly and Company but administered by ABCD.

Otherwise none of the authors have any interests to declare.

## Authors' contributions

JTG came up with the concept for this research project and designed the initial questionnaire. JTG, GAM and EBJ designed the questionnaire with input from the TOPDOC team. JTG, KSR and DW undertook the literature review. JTG and GAM drafted this manuscript, which was revised by DW and DMcG. All authors have read and approved the manuscript.

## TOPDOC Study team

National Steering committee

Dr Gerard A McKay, Dr Edward B Jude, Dr Partha Kar, Dr Barbara McGovan,

Dr Jyothis T George, Dr Jonathan Thow and Prof Stephen L Atkin

Regional Facilitators

Dr Afroze Abbas, Dr Hermione Price, Dr Emma Wilmott, Dr Ragini Bhake, Dr Jeffrin Anthony, Dr David Warriner, Dr Richard Chudleigh, Dr Marc Atkin, Dr Kavitha Rozario, Dr David McGrane, Dr Suma Sugunendran, Dr Arutchelvam Vijayaraman, Dr Prashanth Vas, Dr Mario Shekar, Dr Rahat Ashraf Chaudry, Dr Roselle Herring, Dr Caroline Diacon, Dr Andrew Solomon and Dr Rupa Ahluwalia

Statistical Advisors

Ms. Irene Stratton and Ms. Jackie Cooper

## Pre-publication history

The pre-publication history for this paper can be accessed here:

http://www.biomedcentral.com/1472-6920/10/54/prepub
